# The Multifarious Role of 14-3-3 Family of Proteins in Viral Replication

**DOI:** 10.3390/v12040436

**Published:** 2020-04-13

**Authors:** Kavitha Ganesan Nathan, Sunil K. Lal

**Affiliations:** 1 School of Science, Monash University, Bandar Sunway, Selangor Darul Ehsan 47500, Malaysia; kavitha.r.ganesannathan@monash.edu; 2 Tropical Medicine & Biology Platform, Monash University, Bandar Sunway, Selangor Darul Ehsan 47500, Malaysia

**Keywords:** 14-3-3, host proteins, host–virus interactions, virus life cycle, protein–protein interactions

## Abstract

The 14-3-3 proteins are a family of ubiquitous and exclusively eukaryotic proteins with an astoundingly significant number of binding partners. Their binding alters the activity, stability, localization, and phosphorylation state of a target protein. The association of 14-3-3 proteins with the regulation of a wide range of general and specific signaling pathways suggests their crucial role in health and disease. Recent studies have linked 14-3-3 to several RNA and DNA viruses that may contribute to the pathogenesis and progression of infections. Therefore, comprehensive knowledge of host–virus interactions is vital for understanding the viral life cycle and developing effective therapeutic strategies. Moreover, pharmaceutical research is already moving towards targeting host proteins in the control of virus pathogenesis. As such, targeting the right host protein to interrupt host–virus interactions could be an effective therapeutic strategy. In this review, we generated a 14-3-3 protein interactions roadmap in viruses, using the freely available *Virusmentha* network, an online virus–virus or virus–host interaction tool. Furthermore, we summarize the role of the 14-3-3 family in RNA and DNA viruses. The participation of 14-3-3 in viral infections underlines its significance as a key regulator for the expression of host and viral proteins.

## 1. Introduction

Viruses are a leading source of death and have a significant impact on global health. They are unique pathogens which rely on living organisms to complete their life cycle [1]. Therefore, identifying the viral and host factors involved in viral infection is the main aim of virologists, in order to develop antiviral drugs. Drugs targeting viral proteins have clear disadvantages, such as viral subtype specificity, the rapid development of drug resistance, and low fidelity polymerases in the case of HIV and influenza viruses [2]. Therefore, considering the increasing threat of viral infections, focus is emerging on the need to identify novel, effective, and safe alternative therapies for viral infections. Targeting host proteins is an efficient alternative, since viruses are dependent on host proteins at multiple stages to achieve their replication cycles [3,4,5].

Recently, the 14-3-3 family of proteins has received much attention in the field of disease progression and drug development [6,7,8,9]. The 14-3-3 proteins belong to the highly conserved acidic protein family, which consists of seven mammalian isoforms (beta, epsilon, gamma, eta, sigma, tau, and zeta) [10]. Initially, they were identified in 1967 as abundant brain proteins [11]. In this review, we focus on the 14-3-3 family of proteins and their multifaceted roles in a variety of viral infections.

### 1.1. An Overview of the 14-3-3 Family

Historically, 14-3-3 proteins were named based on their elution and the migration pattern of their two-dimensional DEAE-cellulose chromatography and starch gel electrophoresis [12].

The 14-3-3 proteins are essential regulators of intracellular signaling pathways and upon interacting with their target protein, they modify its function and intracellular localization [13]. Furthermore, they are involved in many processes, including cell-cycle control, cell metabolism, apoptosis, and gene transcription control [10,14,15,16]. Hence, it is vital to understand the role of 14-3-3 during viral infection. In addition, the structure of 14-3-3 provides its great capability to bind to a multitude of functionally diverse signaling proteins. This property allows 14-3-3 proteins to affect multiple pathways in the host cell and thus become the target of a variety of viruses modulating them to alter cellular processes to their advantage.

### 1.2. Structure and General Function of the 14-3-3 Family

The crystal structure of 14-3-3 proteins comprises of highly helical and cup-shaped dimers [17]. The cup-like structure of 14-3-3 dimers is the key to their diverse functions and target binding properties. Moreover, the rigid structure of the 14-3-3 dimer, and the frequent presence of 14-3-3 binding motifs in a single target molecule and within disordered regions, are the standard features which are essential for 14-3-3 proteins to bind to and regulate their binding partners [18]. The 14-3-3 dimers contain nine α-helices, which are organized in an anti-parallel array. Among them, helices αA, αC and αD are involved in dimerization; however, helices αC, αE, αG and αI form a concave amphipathic groove at the ligand-binding site [19,20]. Typically, 14-3-3 proteins bind to phosphorylated serine and threonine residues, with two optimal binding motifs on their targets: RSXpSXP and RXXXpSXP [21]. In some cases, they can also bind to non-phosphorylated targets [21,22].

The general function of 14-3-3 proteins can be classified based on the following characteristics [14,21]: (A) conformational changes of its binding protein: the rigid structure of 14-3-3 proteins may serve as a stable base to support the reshaping of their binding partners and the altering of their activities; (B) masking of a phosphorylated region of a binding protein: 14-3-3 protein binding can also mask significant surface features of the target protein; (C) act as a scaffold molecule or adapter between two other binding proteins [14,21].

Furthermore, various post-translational modifications, such as phosphorylation, acetylation, acylation, polyglycylation and oxidative stress, are documented for 14-3-3 proteins [23,24]. Eventually, these post-translational changes of 14-3-3 isoforms, or their interacting proteins, may have several effects on 14-3-3 proteins, including dimerization specificity, functional regulation, and cellular localization [25]. All these diverse functions of 14-3-3 proteins contribute to an array of protein–protein interactions among the host proteins, and host–virus interactions.

## 2. Host–Virus Interactions

Viruses are a major contributor to the global burden of diseases because they cause acute and chronic infectious diseases [26,27]. Many studies have shown how viruses hijack cellular pathways and evade the host’s innate immune response, starting from entry, until the virus exits from host cells by modulating host factors and signaling pathways [28,29]. Therefore, comprehensive knowledge of host–virus interactions is important in order to understand the viral life cycle and to develop specific therapeutic approaches for the treatment of viral infections. Hence, the study of protein–protein interactions and their communication networks within infected cells has become an important tool for the understanding of the mechanisms of viral replication and, in the long run, leading to the discovery of new anti-viral targets.

### 2.1. Protein–Protein Interactions

Protein–protein interaction (PPI) is one of the primary components of system biology and a key mediator for host–virus interactions. PPI can be detected by in-vitro, in-vivo, and in-silico studies. The in-vitro methods include tandem affinity purification (TAP), co-immunoprecipitation (co-IP), protein fragment complementation, protein arrays, phage displays, X-ray crystallography and nuclear magnetic resonance (NMR) spectroscopy [29,30,31,32]. In contrast, in-vivo methods include yeast two-hybrid (Y2H) [33] and synthetic lethality techniques [34,35]. For in-silico techniques, usually, computer simulations are used to perform sequence- [36] and structure-based predictions [37], gene fusion [38], and in-silico two-hybrid [39], chromosome proximity [40] and gene expression-based approaches [41,42].

### 2.2. Protein–Protein Interaction Network

The interaction between proteins is one of the most critical processes in the execution of cellular processes. In system biology research, protein–protein interaction networking (PPIN) is an essential tool for the identification of interactions between protein pairs. Therefore, the importance of understanding this PPI has led to the development of experimental and computational interaction network tools. As discussed above, a wide variety of methods have been developed to study these interactions.

**Figure 1:**
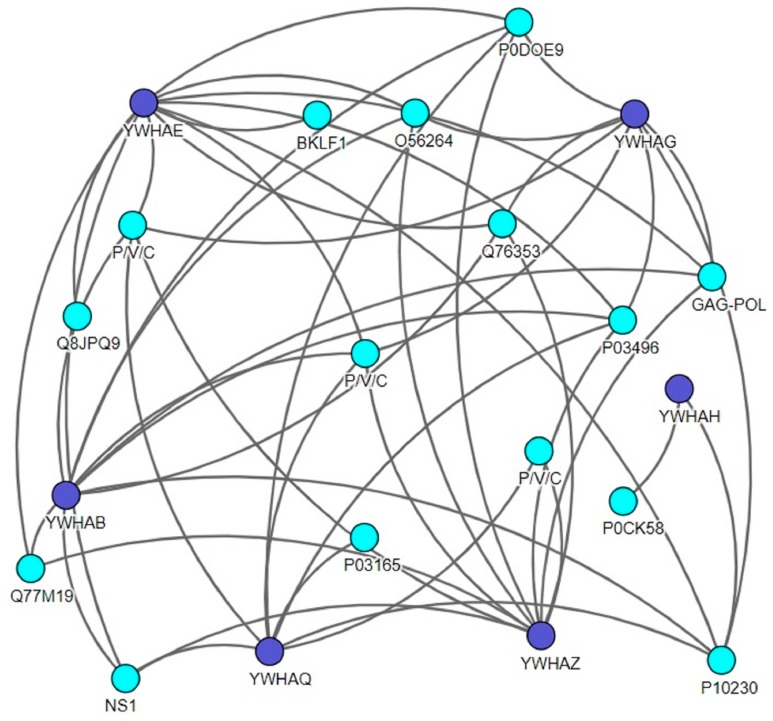
The *Virusmentha* network showing a host (14-3-3s)–virus interaction map. Lines between the proteins indicate interactions between proteins. Purple nodes indicate the different 14-3-3 isoforms, and blue nodes indicate the viral proteins. The *Virusmentha* database is available at https://virusmentha.uniroma2.it/ [44,45].

To date, there are many tools and web-based technologies that allow these data to be explored, providing different methods to visualize the interaction of the protein network. Here, to identify the direct host interaction partners for all viral proteins, we generated a protein–protein interaction map using the *Virusmentha* network (Figure 1). The *Virusmentha* database, which is freely available at https://virusmentha.uniroma2.it/, is the most comprehensive automatically updating resource available to date for viral interactions [42,43].

The isoforms of 14-3-3 proteins, like β, θ, ε, γ, η, and ζ, were found to interact with single-stranded RNA viruses (ssRNA) such as the influenza A virus (IAV), measles virus, human respiratory syncytial virus, human immunodeficiency virus (HIV), La Crosse virus, and double-stranded DNA (dsDNA) viruses like herpes simplex virus type I, human herpes 4, hepatitis B virus (HBV), Nipah virus, Hendra virus and Murid herpesvirus (Table 1). Investigators have confirmed these interactions using different approaches and methods, as described in Table 1. Among all the isoforms of the 14-3-3 family, ζ, ε, and θ are the most established and well-studied in the context of viral infections [46,47,48,49,50,51]. Similarly, co-IP using tandem affinity purification (TAP) and pull-down techniques are the most extensively used methods in the study of protein–protein interactions.

## 3. Role of 14-3-3 Family Members in Virus Infection

The 14-3-3 proteins are associated with many cellular processes, which could probably implicate them in many human illnesses. Therefore, the characterization of their functions at the molecular level is of the utmost importance. Given the multitude of its binding partners and its essential roles in numerous biological processes, the 14-3-3 family of proteins should be considered pathfinders for further exploration of closely related viruses.

Viruses have been shown to arrest host cells by hijacking and manipulating host protein complexes to promote the development of infection. Generally, viruses are classified based on the Baltimore Classification System invented by David Baltimore, which is primarily based on their genome composition and replication strategy [54]. Although there are seven different replication strategies in the system, the primary stages in viral replication are virus attachment, entry, uncoating, transcription, the synthesis of virus components, virion assembly, and the release of progeny virions [55]. However, they are unable to replicate without the machinery and metabolism of a host cell. To further explore the roles of host–virus interaction in the viral life cycle, we discuss the importance of 14-3-3 proteins in RNA and DNA viruses in the subsequent sections.

### 3.1. Role of 14-3-3 Family in RNA Viruses

RNA viruses have ssRNA or dsRNA as their genetic material [56]. In addition, based on the polarity of the RNA, they can be classified into a negative sense or a positive sense [57]. In contrast, dsRNA viruses are a diverse group of viruses that vary widely in their range of hosts (animals, bacteria, plants and fungi), genome segment numbers (one to twelve), and organization of virions (T-number and capsid layers) [58]. Like many viruses, host cells play an essential role in RNA virus replication as well. To date, several isoforms of 14-3-3 proteins are reported to interact with, and play an essential role in, RNA virus infection [59,60,61,62,63,64,65]. Moreover, discussions regarding the 14-3-3 family have dominated research in recent years for their promising progress, which also elucidates their enormous role in controlling cell cycles, apoptosis and signaling pathways.

In 2000, Aoki et al. demonstrated that 14-3-3 proteins associate with the hepatitis C virus (HCV) core protein to activate RAF-1 kinase and contribute to hepatocyte growth regulation [59]. Further, the HCV core protein induces Bax-mediated apoptosis through HCV core–14-3-3 ε protein interaction [60]. In general, the host factor contributes positively to the budding of many negative-stranded RNA viruses, as reviewed elsewhere [66,67]. However, in the case of parainfluenza virus 5 (PIV5), the M protein interacts with host protein 14-3-3 β and negatively affects the production of PIV5 particles [61]. This shows that the interaction of the M protein with 14-3-3 β is not related to virus budding.

The 14-3-3 proteins have a crucial role to play in the regulation of cell cycle progression [10,68]. They regulate cell cycle progression by altering the activities of cell division cycle 25C (Cdc25C), cyclin B1 (a regulatory protein involved in mitosis), checkpoint kinase 1 (Chk1), and wee1 [10,69,70]. The 14-3-3 proteins interact with the accessory protein Vpr (viral protein R) of human immunodeficiency virus type I (HIV-1) and significantly regulate cell cycle progression by associating with Cdc25C [62]. Hence, Vpr induces G2-M cell cycle arrest by increasing the association between 14-3-3 θ and cell cycle regulatory factors (Cdc25C, cyclin B1, and Cdk1) during HIV infection [63]. In addition, Vpr interferes with, and alters the activity of, forkhead transcription factors (Foxo3a) by inhibiting their association with 14-3-3 proteins [71].

Similarly, in the case of adeno-associated virus type 2 (AAV-2) infection, 14-3-3 ε and 14-3-3 γ interact with the Rep68 protein of AAV-2 by phosphorylation at serine 535 [64]. Interestingly, the Rep78 of AAV-2 does not interact with 14-3-3 proteins. Another key point is that the phosphorylation of Rep68 in the C-terminal seven amino acid (LARGHS) of Rep68 is necessary for the interaction of 14-3-3 with Rep68. Moreover, Yaffe et al. described that 14-3-3 proteins’ interaction with target proteins often entails the phosphorylation of a serine residue [11]. Thus, this observation may provide a hint to understanding the role of Rep68 in AAV infections. Furthermore, 14-3-3-Rep68 interaction down-regulates the DNA binding activity of the target protein in an AAV infection [65]. This suggests that 14-3-3 proteins are functionally involved in the regulation of the Rep68 protein during the viral life cycle.

Members of the14-3-3 family regulate numerous intracellular processes, one of which is immunity [72]. Immunity which is 14-3-3-mediated may guide the rational design of therapeutics, and this can be seen in many virus infections [73] as well as mosquito-transmitted Zika and dengue viruses [74,75,76]. In a very recent study, the NS3 protein of the Zika virus interacted with 14-3-3 ε and 14-3-3 η to inhibit RIG-I and the melanoma differentiation-associated protein 5 (MDA5) signaling pathway [75]. Notably, 14-3-3 η was also reported to promote antiviral signaling by MDA5 in HCV infection [77]. In the dengue virus, 14-3-3 ε binds to the NS3 protein and prevents RIG-I from translocating to the mitochondrial antiviral signaling (MAVS) protein adapter, thereby blocking antiviral signaling [76]. Liu et al. reported 14-3-3 ε as a critical mediator for the relocation of RIG-I from cytosol to mitochondrial-associated MAVS, as it establishes a translocon complex with RIG-I and TRIM25, eventually triggering an antiviral response [78].

Previous studies from our laboratory have shown that the 14-3-3 protein also binds to the severe acute respiratory syndrome (SARS) coronavirus nucleocapsid (N) protein [79]. This binding allows the regulation of nucleocytoplasmic shuttling of N protein in a phosphorylation-dependent manner. Additionally, our group has shown that N protein induces apoptosis by reducing the expression level of 14-3-3 θ in the absence of growth factors (serum), which contributes to the accumulation of the phosphorylated N protein in the nucleus [78]. Likewise, coronavirus 2, or COVID-19, originated from China sharing similar demographic profiles, and laboratory and radiological findings with SARS and Middle East respiratory syndrome (MERS) [80]. In SARS, our group has demonstrated that N protein is phosphorylated by multiple kinases (cyclin-dependent kinase, glycogen synthase kinase, and casein kinase II) and significantly interferes with cellular machinery by binding to 14-3-3 proteins through phosphorylation-dependent protein–protein interactions. This suggests that this discovery may provide new insights into a possible mechanism for the phosphorylation-dependent nucleocytoplasmic shuttling of the N protein and could be a potential antiviral strategy against COVID-19.

In addition, a proteomics report showed that the influenza A virus (IAV) NS1 protein interacts with several 14-3-3 isoforms, although the functional implication of this interaction remains unclear [73].

### 3.2. Role of 14-3-3 in DNA Viruses

DNA viruses have DNA as their genetic material and replicate either by host or by virally encoded DNA polymerases [81]. Isoforms of the 14-3-3 family are reported to play essential roles in the life cycle of DNA viruses like the hepatitis B virus (HBV), herpes simplex virus type (HSV-I) and Epstein–Barr virus (EBV) [51,82,83,84]. For instance, in the HBV life cycle, 14-3-3 ζ is responsible for maintaining the HBV protein x (HBx) expression in hepatocellular carcinoma cells (HCC). The knockdown of 14-3-3 ζ reduces the expression of HBx. This suggests that 14-3-3 ζ–HBx interaction could be a potential therapeutic target for HBV-related hepatocellular carcinoma [51]. Over the past few years, research has shown that 14-3-3 proteins are key regulators of many processes, including mitosis and apoptosis in animals [85]. According to Kim et al., HBx induces apoptosis by inhibiting the association between 14-3-3 ε and Bax, thereby enhancing mitochondrial–Bax translocation and cytochrome C release [86]. Consequently, a potential 14-3-3 binding motif of HBx is crucial for stress-activated protein kinases (SAPK)/ Jun amino-terminal kinases (JNK) activitity and Fas-mediated apoptosis protection [87].

**Figure 2:**
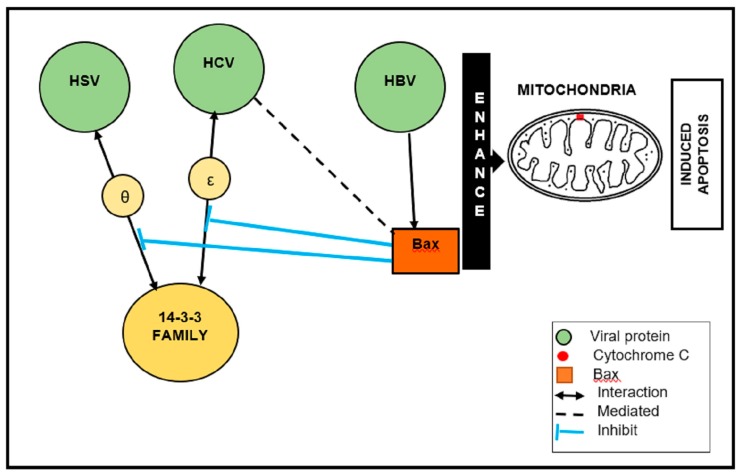
Schematic illustration of the Bax-mediated apoptosis of herpes simplex virus (HBV), hepatitis C virus (HCV), and hepatitis B virus (HBV). HBV, HCV, and HSV directly associate with, or inhibit, cellular protein 14-3-3 to induce apoptosis.

In the case of HSV-I, ICP27 is an essential protein for viral replication because it is involved in the nuclear export of viral mRNA and the suppression of host protein synthesis by inhibiting cellular mRNA splicing [82,83]. ICP27–14-3-3 θ interaction sequesters Bax to the cytoplasm. Further, ICP27 inhibits the interaction between 14-3-3 θ and Bax [82]. It is interesting to note that HBV, HCV, and HSV directly associate with, or inhibit, cellular protein 14-3-3 to induce apoptosis (Figure 2). Typically, (pro-apoptotic) Bax is a member of the Bcl-2 family and a key apoptosis regulator. Upon apoptotic stimulation, Bax is activated and oligomerized in the mitochondrial membrane (MOM) to mediate its permeability [88]. Therefore, 14-3-3 proteins are shown to promote Bax-mediated apoptosis, resulting in Bax being activated and MOM permeabilization.

Gupta et al. identified several members of the 14-3-3 protein family (ζ, ε, γ, β, η) as interacting partners of EBV-encoded large tegument protein deneddylase (BPLF1) using co-IP and mass spectrometry approaches [84]. Interactions between 14-3-3 and BPLF1 may participate in the regulation of many signaling pathways; ubiquitin ligases, cullin 1 (CUL1) and tripartite motif-containing protein 25 (TRIM25) are the possible partners in this regulation. Furthermore, 14-3-3 proteins and TRIM25 serve as vital co-factors in the signaling of viral nucleic acid sensors, such as retinoic acid-inducible gene I (RIG-I) and MDA5 [78]. In this case, 14-3-3 proteins stabilize the interaction of TRIM25 with RIG-I, thus facilitating the ubiquitination of RIG-I [89].

In addition, we also demonstrate and summarize all the roles of 14-3-3 proteins in different viruses (Table 2). The apoptosis pathway, cell cycle, cell signaling, ubiquitin ligase, and nucleocytoplasmic shuttling are the most common pathways which were usurped and manipulated by the viruses to favor their replication.

**Figure 3:**
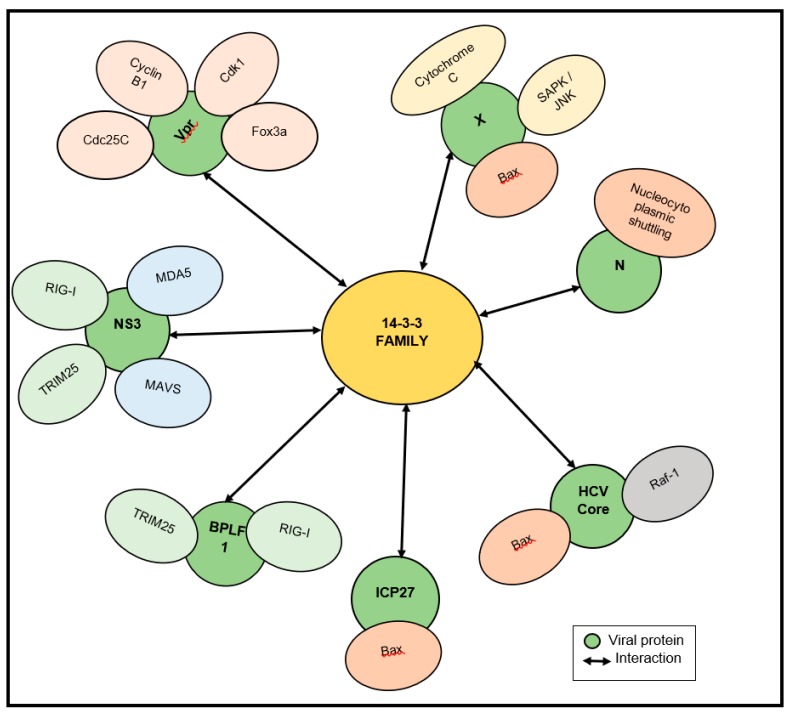
Schematic illustration of the signaling pathways which are manipulated by virus–14-3-3 interactions. Yellow nodes indicate the 14-3-3 family, green nodes indicate the viral proteins and other nodes indicate the functional proteins in signaling pathways.

We also elaborate on all the signaling pathways that are manipulated by 14-3-3–virus interactions (Figure 3). It is interesting to note that cellular proteins are essential for the replication of many RNA and DNA viruses, and may serve as viable targets for treating viral infections.

## 4. Conclusions

Viruses exploit the molecular machinery of the infected host to support their replication. To achieve this, viruses establish virus-specific protein interactions to perturb several cellular processes in the infected host [90,91]. Thus, a comprehensive understanding of the perturbation of the host–virus relationship and virus infection are essential for the development of antiviral therapies. In order to provide an overview of host–virus interactions, we generated a host (14-3-3 proteins)-specific interaction map using *Virusmentha*, an online tool. Based on this database, 14-3-3 ζ, ε, and θ are well known in the context of viral infections.

Furthermore, 14-3-3 family members display a significant role in interacting with several RNA and DNA viruses through multiple pathways, including apoptosis, cell signaling, cell cycle, and ubiquitination. These viral–14-3-3 interactions may change the typical distribution and disrupt the original functions of 14-3-3 proteins. For example, HBV, HCV, and HSV viruses were directly associated with, or inhibited, 14-3-3 proteins from inducing apoptosis. Therefore, monitoring and manipulating 14-3-3 proteins may represent new diagnostic and therapeutic targets for virus infections. The development of therapeutics, including drugs and vaccines, is highly dependent on the knowledge gained from investigating host–virus interactions. As such, interrupting host–virus interactions by targeting the right host factor might be a highly effective strategy for treating viral infections.

## Figures and Tables

**Table 1:** Summary of virus–host protein (14-3-3) interactions using different methods.

*UniProt ID*	Host Protein 14-3-3	Method	Viruses	Target Gene/Protein	Ref
*P03496*	Beta/alpha	TAP	Influenza A Virus (strain A/Puerto Rico/8/1934)	NS	[46]
*Q77M19*	Beta/alpha, zeta, epsilon	Co-IP	Measles virus strain Schwarz	P	[47]
*Q76353*	Beta/alpha, zeta, epsilon	Physical association by using the pull-down, anti-tag co-IP,	Human immunodeficiency virus I (HIV-I)	-	[48]
*P10230*	Eta	Physical association by using Affinity chromatography technology	Herpes simplex virus (type 1 / strain 17)	Tegument protein UL46	[52]
*P03165*	Theta	Physical association by Anti-bait co-IP	HBV ayw/France/Tiollais/1979	X	[49]
*GAG-POL*	Beta/alpha, gamma, epsilon, zeta	Physical association by using the Affinity chromatography technology	HIV -I	Gag-pol	[48]
*P0DOE9*	Beta/alpha, zeta, epsilon, gamma, theta	co-IP	Human respiratory syncytial virus A2	1C	[50]
*P0CK58*	Eta	TAP	Human herpesvirus 4 (strain B95-8)	Apoptosis regulator BALF1	[53]
*Q8JPQ9*	Epsilon	TAP	La Crosse virus L78	N	[46]
*Q997F2*	Theta, zeta	TAP	Nipah virus	P/V/C	[46]
*P0C1C6*	Gamma, theta, epsilon, beta/alpha, zeta	TAP	Hendra virus horse/Australia/Hendra/1994	P/V/C	[46]
*P0C1C7*	Gamma, theta, epsilon, beta/alpha, zeta	TAP	Nipah virus	P/V/C	[46]
*Q9WPI5*	Theta	TAP	IAV (A/Texas/36/1991(H1N1))	NS1	[46]
*P88993*	Epsilon	Physical association by using the Two-hybrid array	Murid herpesvirus 4	BKLF1	[51]

**Table 2:** Summary of the 14-3-3 family’s roles in different viruses.

14-3-3	Genome	Viruses	Target Protein	Pathway/Function	Ref
ζ, ε	dsDNA	Hepatitis B virus	Protein x	Bax-mediated apoptosis	[86]
ε	(+) *ssRNA*	Hepatitis C virus	HCV core	Bax-mediated apoptosis	[60]
θ	dsDNA	Herpes simplex virus type I	ICP27	Bax-mediated apoptosis	[82,88]
β	(−) ssRNA	Parainfluenza virus 5	M protein	Virus budding	[61]
θ	(+) ssRNA	Human immunodeficiency virus type I	Vpr	Cell cycle	[62,63]
ε, γ	ssDNA	Adeno-associated virus type 2	Rep68	Virus replication	[65]
ζ, ε, γ, β, η	dsDNA	Epstein-Barr virus	BPLF1	Cell signaling and ubiquitin ligase	[78,89]
ε, η	(+) *ssRNA*	Zika virus	NS3	Cell signaling and ubiquitin ligase	[75]
θ	(+) ssRNA	Coronavirus	N	Nucleocytoplasmic shuttling	[78,79]
